# Stability Study of Cervical Specimens Collected by Swab and Stored Dry Followed by Human Papillomavirus DNA Detection Using the cobas 4800 Test

**DOI:** 10.1128/JCM.02025-16

**Published:** 2017-01-25

**Authors:** Chun-Qing Lin, Xi Zeng, Jian-Feng Cui, Guang-Dong Liao, Ze-Ni Wu, Qian-Qian Gao, Xun Zhang, Xiu-Zhang Yu, Wen Chen, Ming-Rong Xi, You-Lin Qiao

**Affiliations:** a Department of Cancer Epidemiology, Cancer Hospital, Chinese Academy of Medical Sciences and Peking Union Medical College, Chaoyang District, Beijing, China; b Department of Gynecology and Obstetrics, West China Second University Hospital, Sichuan University, Key Laboratory of Birth Defects and Related Diseases of Women and Children, Ministry of Education, Chengdu, Sichuan, China; c Department of Pathology, Cancer Hospital, Chinese Academy of Medical Sciences and Peking Union Medical College, Chaoyang District, Beijing, China; Rhode Island Hospital

**Keywords:** dry storage, human papillomavirus, stability, swab

## Abstract

Safer, more convenient methods for cervical sample collection and storage are necessary to facilitate human papillomavirus (HPV) DNA testing in low-resource settings. Our study aimed to evaluate the stability of cervical specimens collected with dry swabs and stored dry, compared to liquid-based cytology (LBC) samples, as detected by HPV DNA testing. Women with abnormal cytological findings or HPV-positive results at colposcopy were recruited from the West China Second University Hospital, Sichuan University, between October 2013 and March 2014. From each woman, physicians collected cervical specimens with a swab placed into a Sarstedt tube and a CytoBrush placed into LBC medium. Samples were randomly assigned to be stored at uncontrolled ambient temperature for 2, 7, 14, or 28 days and then were tested for 14 high-risk HPV (HR-HPV) types using the cobas HPV test. The rates of agreement between dry swab and LBC samples for any HR-HPV type, HPV16, HPV18, and the 12 pooled HR-HPV types were 93.8%, 97.8%, 99.4%, and 93.2%, respectively, with kappa values of 0.87 (95% confidence interval [CI], 0.83 to 0.91), 0.94 (95% CI, 0.91 to 0.97), 0.94 (95% CI, 0.87 to 1.00), and 0.86 (95% CI, 0.82 to 0.90). The performance of swab samples for detection of cervical precancerous lesions by means of cobas HPV testing was equal to that of LBC samples, even with stratification by storage time. Dry storage of swab-collected cervical samples can last for 1 month without loss of test performance by cobas HPV testing, compared to LBC samples, which may offer a simple inexpensive approach for cervical cancer screening in low-resource settings.

## INTRODUCTION

Human papillomavirus (HPV) DNA testing has proven to be an effective primary screening approach for the secondary prevention of cervical cancer in developing countries (1, 2). The assays for detecting HPV offer important advantages, including greater reliability and easier implementation, because these molecular tests do not require specialized medical training to obtain samples (3, 4). Currently, however, most HPV detection assays rely on liquid-based cytology (LBC) medium. In low-resource settings, where the use of Pap testing is limited, LBC medium adds cost, is difficult to transport, and represents a waste disposal challenge. Therefore, safer and more convenient methods of specimen collection and transport that do not compromise test performance are needed. In the present study, we aimed to investigate the stability of cervical specimens collected by swab and stored dry at ambient room temperature, using HPV DNA testing.

## RESULTS

Specimens were collected from 743 women; 695 women were included for data analysis, however, because there were 48 ineligible cases. Two LBC samples and four swab samples were tested but yielded invalid results. There were 30 cases with no biopsy results among 689 women with valid cobas HPV results. A total of 689 cases were used for agreement analysis, and 659 cases were used to analyze the accuracy of swab and LBC samples for detection of cervical intraepithelial neoplasia grade 2 or worse (CIN2+) and CIN grade 3 or worse (CIN3+) with the cobas HPV test. Characteristics of the 695 eligible women are presented in Table 1.

**TABLE 1:** Characteristics of 695 eligible women

Characteristic	No. (%)
Age	
<35 yr	220 (31.7)
35–44 yr	262 (37.7)
≥45 yr	213 (30.7)
Reason for colposcopy	
Abnormal cytological or HPV-positive results	654 (94.1)
Other reasons	41 (5.9)
Colposcopy result	
Normal	30 (4.3)
Abnormal	658 (94.7)
Missing	7 (1.0)
Biopsy result	
Negative^*a*^	312 (44.9)
CIN1	108 (15.5)
CIN2	47 (6.8)
CIN3	150 (21.6)
Invasive cervical cancer	48 (6.9)
Storage time	
2 days	168 (24.2)
7 days	178 (25.6)
14 days	175 (25.2)
28 days	174 (25.0)
Total	695 (100.0)

a Normal or non-CIN/cervical cancer.

The prevalence of high-risk HPVs (HR-HPVs) overall, 12 pooled HR-HPVs, HPV16, and HPV18, as detected by cobas HPV testing with LBC and swab samples, is shown in Table 2. There were no significant differences in the distributions of HR-HPVs overall, 12 pooled HR-HPVs, HPV16, and HPV18 between LBC and swab samples, with *P* values of 0.61, 0.33, 0.73, and 0.91, respectively. There was also no significant difference in the distribution of invalid cases between LBC and swab samples (*P* = 0.41).

**TABLE 2:** Prevalence for HR-HPVs overall, 12 pooled HR-HPVs, HPV16, and HPV18 by cobas 4800 HPV testing with LBC and swab samples

HPV type	Prevalence (% [no. positive/total no. tested])		*P*
	LBC samples	Swab samples	
HR-HPVs overall	61.9 (429/693)	63.2 (437/691)	0.61
12 pooled HR-HPVs	42.9 (297/693)	45.4 (314/691)	0.33
HPV16	22.4 (155/693)	23.2 (160/691)	0.73
HPV18	4.9 (34/693)	4.8 (33/691)	0.91
Invalid result	0.3 (2/695)	0.6 (4/695)	0.41

The kappa values for agreement between findings for LBC and swab samples detected by cobas HPV testing, stratified according to storage time, are presented in Table 3. The kappa values for HR-HPVs overall, 12 pooled HR-HPVs, HPV16, and HPV18 in LBC and swab samples were 0.87 (95% confidence interval [CI], 0.83 to 0.91), 0.86 (95% CI, 0.82 to 0.90), 0.94 (95% CI, 0.91 to 0.97), and 0.94 (95% CI, 0.87 to 1.00), respectively. The numbers of HR-HPVs overall, 12 pooled HR-HPVs, HVP16, and HPV18 detected in LBC and swab samples by cobas HPV testing agreed well at storage times of 2, 7, and 28 days; however, the storage time of 14 days showed a slightly lower kappa value (0.76 [95% CI, 0.66 to 0.86]) for HR-HPVs overall, compared to that at 7 days (0.93 [95% CI, 0.87 to 0.98]). The sensitivity and specificity for detecting CIN2+ and CIN3+ by cobas 4800 HPV testing using LBC and swab samples are shown in Table 4. There was no significant difference in the sensitivity and specificity for detecting CIN2+ and CIN3+ with these two sample types using the cobas HPV test, regardless of storage time.

**TABLE 3:** Agreement between LBC and swab samples tested by the cobas 4800 HPV test

Storage time and HPV type	No.				Agreement rate (% [95% CI])	Kappa (95% CI)
	LBC sample negative and swab sample negative	LBC sample negative and swab sample positive	LBC sample positive and swab sample negative	LBC sample positive and swab sample positive		
Total (*n* = 689)						
HR-HPVs overall	235	26	17	411	93.8 (91.7–95.3)	0.87 (0.83–0.91)
12 pooled HR-HPVs	360	32	15	282	93.2 (91.1–94.8)	0.86 (0.82–0.90)
HPV16	524	10	5	150	97.8 (96.4–98.7)	0.94 (0.91–0.97)
HPV18	654	2	2	31	99.4 (98.5–99.8)	0.94 (0.87–1.00)
Storage for 2 days (*n* = 168)						
HR-HPVs overall	65	6	3	94	94.6 (90.1–97.2)	0.89 (0.82–0.96)
12 pooled HR-HPVs	89	8	5	66	92.3 (87.2–95.4)	0.84 (0.76–0.92)
HPV16	135	1	0	32	99.4 (96.7–99.9)	0.98 (0.94–1.00)
HPV18	163	0	0	5	100.0 (97.8–100.0)	1.00 (1.00–1.00)
Storage for 7 days (*n* = 176)						
HR-HPVs overall	59	4	2	111	96.6 (92.8–98.4)	0.93 (0.87–0.98)
12 pooled HR-HPVs	92	7	2	75	94.9 (90.6–97.3)	0.90 (0.83–0.96)
HPV16	132	3	1	40	97.7 (94.3–99.1)	0.94 (0.88–1.00)
HPV18	165	0	1	10	99.4 (96.9–99.9)	0.95 (0.85–1.00)
Storage for 14 days (*n* = 174)						
HR-HPVs overall	61	12	8	93	88.5 (82.9–92.4)	0.76 (0.66–0.86)
12 pooled HR-HPVs	94	11	4	65	91.4 (86.3–94.7)	0.82 (0.74–0.91)
HPV16	131	2	4	37	96.6 (92.7–98.4)	0.90 (0.83–0.98)
HPV18	167	2	1	4	98.3 (95.1–99.4)	0.72 (0.41–1.00)
Storage for 28 days (*n* = 171)						
HR-HPVs overall	50	4	4	113	95.3 (91.0–97.6)	0.89 (0.82–0.96)
12 pooled HR-HPVs	85	6	4	76	94.2 (89.6–96.8)	0.88 (0.81–0.95)
HPV16	126	4	0	41	97.7 (94.1–99.1)	0.94 (0.88–1.00)
HPV18	159	0	0	12	100.0 (97.8–100.0)	1.00 (1.00–1.00)

**TABLE 4:** Sensitivity and specificity for detecting CIN2+ and CIN3+ using cobas 4800 HPV testing with LBC and swab samples

Storage time and sample type	CIN2+		CIN3+	
	Sensitivity (% [95% CI])	Specificity (% [95% CI])	Sensitivity (% [95% CI])	Specificity (% [95% CI])
Total (*n* = 659)				
LBC	91.4 (87.2–94.3)	53.7 (48.9–58.5)	92.9 (88.4–95.7)	49.8 (45.2–54.3)
Swab	93.4 (89.6–95.9)	52.8 (48.0–57.5)	93.9 (89.7–96.5)	48.3 (43.7–52.8)
Storage for 2 days (*n* = 161)				
LBC	89.5 (78.9–95.1)	56.7 (47.1–65.9)	89.6 (77.8–95.5)	53.1 (44.0–62.0)
Swab	89.5 (78.9–95.1)	55.8 (46.2–64.9)	89.6 (77.8–95.5)	52.2 (43.1–61.2)
Storage for 7 days (*n* = 170)				
LBC	96.7 (88.8–99.1)	53.2 (43.9–62.3)	98.0 (89.5–99.7)	49.2 (40.4–58.0)
Swab	98.4 (91.3–99.7)	51.4 (42.1–60.6)	98.0 (89.5–99.7)	46.7 (38.0–55.6)
Storage for 14 days (*n* = 164)				
LBC	86.7 (75.8–93.1)	57.7 (48.1–66.8)	91.1 (79.3–96.5)	53.8 (44.9–62.5)
Swab	93.3 (84.1–97.4)	56.7 (47.1–65.9)	95.6 (85.2–98.8)	51.3 (42.4–60.1)
Storage for 28 days (*n* = 164)				
LBC	92.4 (83.5–96.7)	46.9 (37.4–56.8)	92.6 (82.5–97.1)	42.7 (33.9–52.1)
Swab	92.4 (83.5–96.7)	46.9 (37.4–56.8)	92.6 (82.5–97.1)	42.7 (33.9–52.1)

## DISCUSSION

The performance in detecting HR-HPVs overall, 12 pooled HR-HPVs, HPV16, and HPV18 with dry-stored swab samples, which were stored at uncontrolled ambient temperatures for 1 month, was equal to that with LBC specimens using the cobas HPV test. The accuracy of detecting CIN2+ or CIN3+ with dry-stored swab samples with 1 month of storage was comparable to that with LBC specimens. Our study also showed a common limitation of the HPV test, i.e., low specificity for detecting cervical precancerous lesions, with both LBC and swab samples. In this respect, genotyping of HPV16 and HPV18 with the cobas HPV test can compensate for the loss of specificity (5). Of note, although the kappa value for HR-HPVs overall with the two sample types at a storage time of 14 days was slightly lower than that at 7 days, with marginal significance, no significant difference was seen in terms of accuracy in detecting CIN2+ and CIN3+ using the cobas HPV test with LBC and swab samples. The results demonstrated excellent agreement between LBC and swab samples for detection of HR-HPVs overall, 12 pooled HR-HPVs, HPV16, and HPV18 with the cobas HPV test.

Several studies demonstrated that the testing performance of LBC samples did not decline over time (6–8). In the current study, commonly used LBC samples were selected as a reference to evaluate the stability of swab samples. A series of studies compared the performance of dry and wet cervicovaginal samples in HPV assays, but the stability of the two sample types was not evaluated (9–15). Our study is the first, to the best of our knowledge, to demonstrate the stability of dry and wet cervical samples for HPV DNA testing, but we did not evaluate the performance of vaginal or self-collected samples in cobas HPV testing by means of the current dry-collected and dry-stored approach. Studies indicate that the strategy of self-collecting samples for HPV testing is effective in improving cervical screening coverage (16, 17). PCR-based HPV DNA tests have shown similar sensitivities with self-collected samples and clinician-collected samples (18). Whether cobas HPV testing can facilitate the approach of dry and self-collected sampling in cervical cancer screening programs should be further investigated.

The limitations of the current study are as follows. First, the accuracy of dry sample collection for detecting HPV and cervical precancerous lesions, compared to the liquid-based sampling strategy, was evaluated among patients at a colposcopic clinic rather than in the general female population. Second, the processing time for dry-collected and stored swab samples exceeded that of routine methods. The feasibility of shorter periods of vortex-mixing for preparation of dry swab samples should be further tested. In conclusion, swab-collected samples can last up to 1 month in dry storage without loss of test performance, compared to traditional LBC samples, which may offer a simple inexpensive approach of sampling in cervical cancer screening programs in low-resource settings.

## MATERIALS AND METHODS

### Study population.

A parallel comparative experimental study was conducted in the Cancer Hospital, Chinese Academy of Medical Sciences, collaborating with the West China Second University Hospital, Sichuan University. Patients with abnormal cytological findings and/or HPV-positive results were recruited at the time of the colposcopy examination. All women supplied written informed consent. We excluded participants who were pregnant or had a history of total hysterectomy.

### Sample collection and storage.

Two cervical specimens from each woman were randomly collected by the physician prior to colposcopic evaluation. One sample was collected using a CytoBrush and placed into PreservCyt medium (Hologic, Crawley, United Kingdom) (LBC sample), and one sample was collected using a polyethylene fiber swab and placed into a Sarstedt 15-ml tube (swab sample). The swab and Sarstedt 15-ml tube were provided by Roche Molecular Systems (Branchburg, NJ). The specimens were randomly assigned to be stored at uncontrolled ambient temperatures (0 to 30°C) for fixed times of 2, 7, 14, or 28 days. After storage at room temperature according to the protocol, the specimens were stored at −20°C until HPV DNA testing was performed. The specimens were stored at −20°C from 3 months to 6 months before HPV DNA testing was performed.

### Colposcopy and biopsy.

Women classified as having a cervical abnormality by digital colposcopy underwent a biopsy. All pathology slides were read by the pathologists at the West China Second University Hospital, Sichuan University. Ten percent of the CIN1 and CIN3 cases (selected randomly) and all CIN2 cases were subjected to review by the pathologists at the Cancer Hospital, Chinese Academy of Medical Sciences, for quality control.

### HPV DNA testing.

The cobas HPV testing was performed in the laboratory of the West China Second University Hospital, Sichuan University, according to the manufacturer's instructions, except as noted below. The cobas HPV test provides specific genotyping results for HPV16 and HPV18 along with results for 12 pooled oncogenic types, i.e., HPV31, HPV33, HPV35, HPV39, HPV45, HPV51, HPV52, HPV56, HPV58, HPV59, HPV66, and HPV68. Specimen preparation for the cobas HPV test was accomplished using the cobas x 480 instrument, which simultaneously extracts, purifies, and prepares target HPV DNA and β-globin DNA for PCR amplification and detection. The processing of β-globin DNA functions as a control to differentiate HPV-negative specimens from samples that fail to exhibit positivity due to a lack of cells or the presence of PCR inhibitors in the specimens being tested. For the dry swab specimens, we added 1.2 ml PreservCyt medium into the tube with the dry swab specimen and then vortex-mixed the sample for 15 min. After vortex-mixing, the swab was removed from the tube. The tube with the sample eluted from the dry swab was then placed in the cobas 4800 instrument for DNA extraction and HPV detection.

### Statistical analysis.

From PASS software, a sample size of 168 pairs achieved 80% power to detect an odds ratio of 3.00 using a two-sided McNemar test, with a significance level of 0.05. We evaluated the agreement of results for HR-HPVs overall, HPV16, HPV18, and 12 pooled HR-HPV strains detected by cobas HPV testing with swab and LBC specimens for each storage time (2, 7, 14, and 28 days), by means of McNemar tests. We calculated the sensitivity and specificity for detecting CIN2+ and CIN3+ using cobas HPV testing with LBC and swab samples. All *P* values of <0.05 (two-sided) were considered statistically significant. The statistical analysis was conducted using SAS version 9.2 (SAS Institute, Cary, NC).
